# Preparation of Copper-Based Catalysts for Obtaining Methanol by the Chemical Impregnation Method

**DOI:** 10.3390/ma17040847

**Published:** 2024-02-09

**Authors:** Anisoara Oubraham, Mihaela Iordache, Elena Marin, Claudia Sisu, Simona Borta, Amalia Soare, Catalin Capris, Adriana Marinoiu

**Affiliations:** National Institute for Cryogenics and Isotopic Technologies—ICSI Ramnicu Valcea, Uzinei Str. No. 4, 240050 Ramnicu Valcea, Romania; anisoara.oubraham@icsi.ro (A.O.); elena.marin@icsi.ro (E.M.); claudia.sisu@icsi.ro (C.S.); simona.borta@icsi.ro (S.B.); amalia.soare@icsi.ro (A.S.); catalin.capris@icsi.ro (C.C.); adriana.marinoiu@icsi.ro (A.M.)

**Keywords:** copper catalyst, impregnation method, catalytic, methanol, precursor

## Abstract

This paper presents the preparation of heterogeneous catalysts for the direct hydrogenation process of CO_2_ to methanol. The development of the modern chemical industry is inextricably linked to the use of catalytic processes. As a result, currently over 80% of new technologies introduced in the chemical industry incorporate catalytic processes. Since the basic factor of catalytic processes is the catalysts, the studies for the deepening of the knowledge regarding the nature of the action of the catalysts, for the development of new catalysts and catalytic systems, as well as for their improvement, represent a research priority of a fundamental or applied nature. The Cu/ZnO/Al_2_O_3_ catalyst for the synthesis of green methanol, using precursors of an inorganic (copper nitrate, denoted by Cu/ZnO/Al_2_O_3_-1) and organic (copper acetate, denoted by Cu/ZnO/Al_2_O_3_-2) nature, are obtained by chemical impregnation that includes two stages: preparation and one of calcination. The preparation methods and conditions, as well as the physico-chemical properties of the catalyst precursor, play a major role in the behavior of the catalysts. The prepared catalysts were characterized using atomic adsorption analysis, scanning electron microscopy (SEM) with energy dispersive X-ray (EDX) analysis, specific surface area and pore size analyses, adsorption, and the chemisorption of vapor (BET).

## 1. Introduction

Carbon dioxide (CO_2_) is one of the greenhouse gases that cause global warming. Currently, most of the world’s energy comes from burning fossil fuels (coal, oil, and natural gas). The primary energy sources with the most negligible CO_2_ emissions are wind energy, solar energy, hydroelectric energy, ocean energy, geothermal energy, biomass, and biofuels. Regarding the control of CO_2_ emissions, it has been the subject of extensive research. The use of CO_2_ as a non-polluting and renewable carbon source has attracted much attention worldwide, leading to major progress in the energy-efficient catalytic conversion of CO_2_ [1,2]. Carbon capture, use, and storage technologies consist of a set of proposed technological solutions (i.e., methods, measures, implementations, and policies) that aim to capture carbon dioxide before its release into the atmosphere. Carbon dioxide is the main form of the carbon-carrying molecule responsible for the greenhouse effect, which originates from human economic activities and the destabilization of the planetary climate. Climate change and the unsustainability of fossil fuels are calling for cleaner energies, such as methanol as a fuel. Compared to fossil fuels, burning biomethanol reduces nitrogen oxide emissions by up to 80% and carbon dioxide emissions by up to 95% and eliminates sulfur oxide emissions. Methanol has several properties that make it an attractive candidate as an alternative fuel. One of these properties is a high octane number; methanol has a high-octane rating, which means it can better withstand compression in internal combustion engines. This allows for better performance and greater efficiency compared to conventional fuels. Another property is low emissions; ethanol produces low emissions of greenhouse gases and air pollutants compared to fossil fuels. Burning methanol generates carbon dioxide (CO_2_), but this can be captured and used for other purposes, such as the production of plastics or synthetic fuels. A third property is methanol biodegradability; unlike other fuels, methanol is biodegradable and does not persist in the environment for long. This makes it a safer option and less damaging to ecosystems [2]. The benefits are two-fold: the process removes carbon dioxide from the atmosphere and turns it into an alternative fuel to current crude oil-based fuels. This is an important step and could even lead to the creation of a “methanol economy” in the future, just as the era of industrialization was based on coal, and now crude oil is used. The cost and yield of biomethanol are highly dependent on the characteristics of the feedstock, the initial investment, and the facility. The researchers studied the environmental analysis of using renewable methanol as a fuel instead of conventional fossil fuels in the shipping industry from a technical point of view without major challenges in managing the potential supply chain to reduce emissions from shipping. [3]. Power-to-liquid (PtL) systems represent a viable solution for future energy scenarios. PtL technology refers to a process that is able to absorb energy and convert and store surplus energy from renewable sources in the grid in a chemical form. Due to the importance of catalytic processes in the chemical industry and the continuous need for them to be improved in order to reduce environmental pollution and also to reduce costs, many efforts are being made to develop new synthesis routes that allow the improvement of physical properties, the chemistry of the catalysts, and to maximize their activities [4,5,6]. Hybrid materials with suitable porosity can be successfully used in catalysis, optics, membrane technology, and biomimetics. Further, hybrid materials present advantages in the field of catalysis regarding the capture and conversion of CO_2_ in order to reduce environmental pollution and transform it into products with added value [7,8]. One of the current concerns in the field of catalysis and nanotechnology is the development of catalysts that can be easily tailored according to the industrial application in which they are used [9]. The thermo-catalytic hydrogenation of CO_2_ to obtain CH_3_OH by heterogeneous catalysis is one of the promising approaches that have attracted much attention in recent research [10,11,12]. Major advances have been made in the development of various catalysts, including metals, metal oxides, and intermetallic compounds [6,7,8]. The synthesis of green methanol, starting from pure sources and controlled concentrations of CO_2_ and H_2_, greatly simplifies the chemistry and reaction products. The chemical reaction to obtain methanol is shown below:

CO_2_ +3H_2_ ⇋ CH_3_OH +H_2_O + heat (1)

This process represents an ecological solution for methanol production, allowing a reduction in the intensity of fossil fuel consumption and mitigating CO_2_ emissions [13,14,15,16]. The catalyst is often, if not always, the “center piece” of the chemical process, and the strategy chosen in its preparation technique is the most important step in understanding the catalyst’s production [17,18]. The state-of-the-art copper-based catalyst for the synthesis of methanol from CO_2_ has the following formula: Cu/ZnO/Al_2_O_3_. In an analogy with methanol synthesis from syngas, the active sites for CO_2_ hydrogenation in conventional copper-based catalysts are bound to partially or fully reduced Cu with synergistic contact with partially reduced ZnO or ZnO_x_ (zinc oxide) [19,20]. ZnO plays an important role in the performance of catalysts, as it favors the dispersion and stabilization of Cu active sites and facilitates the adsorption of CO_2_ to be subsequently hydrogenated in methanol. In the case of Al_2_O_3_ (aluminum oxide), this oxide improves the exposure and stabilization under reaction conditions of the Cu-active centers. Metal oxides are binary compounds or mixtures of elements with oxygen [21,22,23]. Most metal oxides are ionic lattice materials. They represent one of the most important and widely used categories of solid catalysts, either as active phases or as supports. In both crystalline and amorphous structures, the use of these metal oxides in catalysis is linked to both acid-base and redox properties. They represent a very important family of catalysts in heterogeneous catalysis. Compared to alternative noble metals, they have the advantage of being cheap and reliable. However, the chemistry of metal oxides is not straightforward, especially due to the surface contribution [15,24]. Supported metal oxides are used as environmental catalysts to selectively transform unwanted environmental pollutants into non-harmful forms [25]. Supported catalysts have a very general design of solid catalysts, in which the active phase is present on the surface of a solid, in this case, the support. Obviously, the dispersion and stabilization of the support layer on a support with a large specific surface lead to catalysts in the form of a composite material, extremely active and robust [26,27,28]. The main characteristics of the supports are as follows: (a) providing a large surface area, separating the active phase, and preventing the formation of large crystalline particles; and (b) providing the space in which the catalytic reactions take place [29,30].

The preparation techniques of catalysts in the field of chemistry are the following: impregnation, co-precipitation, and sol-gel [31,32]. The method chosen to prepare the catalysts in this study was the impregnation method. Impregnation is the procedure by which a certain volume of solution containing the precursor of the active phase is brought into contact with the solid phase, which, in a subsequent step, is dried to remove the solvent from the mixture [33,34,35]. Thus, this method can be used to prepare supported and mixed catalysts [36]. Once the catalyst is impregnated on the support or other active solid phase, it is then dried and calcined or reduced. The advantage of this method is the short reaction time. The disadvantage of this method is the fact that it is more difficult to obtain catalysts with a high concentration and a uniform dispersion of the catalyst components on the surface. However, the wet impregnation method is much more advantageous compared to other methods used in the preparation of catalysts; namely, it is easy to prepare a layer of active material on the surface of the catalyst [37,38,39]. The preparation methods and conditions, as well as the physico-chemical properties of the precursor used, play an important role in the behavior of catalysts in chemical reactions.

In this work, heterogeneous catalysts will be developed for the direct hydrogenation process of CO_2_ to methanol. The main effort is focused on the improvement of conventional Cu/ZnO/Al_2_O_3_ catalysts and the development of new catalytic systems targeting the specific needs for CO_2_ to methanol reactions. Major studies on the development of active and selective catalysts, based on thermodynamics, mechanisms, nano synthesis, and catalyst design (active phase, promoters, supports, etc.), are highlighted. Thus, in this study, two types of catalysts were prepared using two types of precursors (one inorganic and one organic). These two catalysts were compared with a commercial catalyst purchased from BASF (Badische Anilin- und Soda-Fabrik, Ludwigshafen, Germany). The chosen method was impregnation due to the advantages it presents. The catalysts were characterized by several physico-chemical methods. The aim of the work is to obtain copper-based catalysts for the production of methanol. The obtained methanol can be used in fuel cells with direct methanol supply. The advantages of the file of fuel with direct methanol feed are low cost, wide scale of use, and safe storage. The novelty and relevance of this research derive from the use of two metal precursors to obtain an alternative catalyst to the commercially available one (BASF).

## 2. Materials and Methods

### 2.1. Reagents

The synthesis reagents of analytical purity were used without further purification. The materials used for the preparation of copper-based catalysts were as follows: copper acetate (CH_3_-COO)_2_Cu ·H_2_O, 99.5%, S.C UTCHIM SRL, Ramnicu Valcea, Romania); copper nitrate (Cu (NO_3_)_2_·2.5 H_2_O, 98.0%, Alfa Aesar, MA, USA), zinc oxide (ZnO, 99.0%, Alfa Aesar, MA, USA), aluminum oxide (Al_2_O_3_, 99.97%, Alfa Aesar, MA, USA), and distilled water. The Millipore system (Stuttgart, Germany) was used to purify the water.

### 2.2. Synthesis

**Figure 1:**
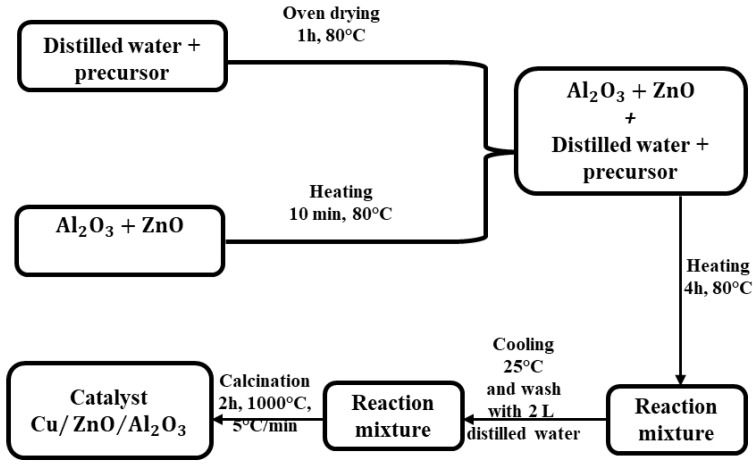
Summarized scheme for the Cu/ZnO/Al_2_O_3_ powder catalyst synthesis using the impregnation method.

The Cu/ZnO/Al_2_O_3_ catalyst is of particular interest for the direct hydrogenation process of CO_2_ to methanol. The main effort is focused on the improvement of conventional Cu-based catalysts, starting from the inorganic precursor (copper nitrate) and organic (copper acetate), and thus developing two types of catalysts targeting the specific needs for CO_2_ to methanol reactions. These copper precursors were chosen because they present the most convenient scrubbing method considering the formation of CO_2_ and NO_2_ and their removal. The chemical impregnation method was used as a preparation technique. The Cu/ZnO/Al_2_O_3_ catalyst was prepared as follows: 5 g of aluminum oxide (Al_2_O_3_) and 20 g of zinc oxide (ZnO) were dried for 1 h in an oven at a temperature of 80 °C. Ground and sieved aluminum oxide was used. Further, 40 g of each precursor mixed with distilled water was kept on the stove for ten minutes at a temperature of 80 °C, after which the zinc and aluminum oxides were added, and the reaction mixture obtained was left— -stirring for 4 h at 80 °C. After the reaction mixture had cooled, it was washed with 2 L of distilled water. The powder was calcined at 1000 °C for 2 h at a heating rate of 5 °C min^−1^ in a nitrogen atmosphere in a tube furnace to remove any volatile chemical species. The calcination temperature was chosen at 1000 °C because it must be below the melting temperature of the substances in the catalyst structure. Therefore, it was chosen below the melting temperature of copper (the melting point of copper is 1085 °C). Figure 1 summarizes the Cu/ZnO/Al_2_O_3_ catalyst powder synthesis using the impregnation method. The preparation method of the catalysts is presented in Table 1.

## 3. Results

### 3.1. Determination of Cu and Zn Concentrations by Flame Atomic Absorption Spectrophotometry Method

The Cu–Zn content was determined by flame atomic absorption spectrophotometry using a NOVAA 300 atomic absorption spectrophotometer (Analytik Jena, Jena, Germany) equipped with a deuterium lamp for background correction. It allows the sequential analysis of metallic and non-metallic traces in liquid and dissolved samples. All chemicals and reagents used during the study were of spectroscopic grade; a CertiPur multielement standard solution with a certified value of 1000 mg/l obtained from Merck (Darmstadt, Germany) was used for the calibration curve. All investigated calibration curves were characterized by a high correlation coefficient (r > 0.995). The analyses were performed in an air-acetylene flame, and the radiation sources used were lamps with a cavity cathode corresponding to each analyzed element. A TOPWAVE microwave extraction system (Ramsey Road, Shirley, NY, USA) was used for sample preparation/mineralization, equipped with 12 digestion/mineralization vessels with sensors for temperature and pressure control in the vessels on the rotor (Table 2 and Table 3). After the digestion process with aqua regia (7 mL of azotic acid (HNO_3_) conc. 65%, and 21 mL of hydrochloric acid (HCl) conc. 37%), each extract was transferred quantitatively with ultrapure water in a 50 mL volumetric flask. The Cu and Zn elements from the catalyst samples were analyzed according to the standard methods in force. The results of the samples obtained by flame atomic absorption spectrometry are presented in Table 4.

### 3.2. Determination of Particle Size by Dynamic Light Scattering Method

**Figure 2:**
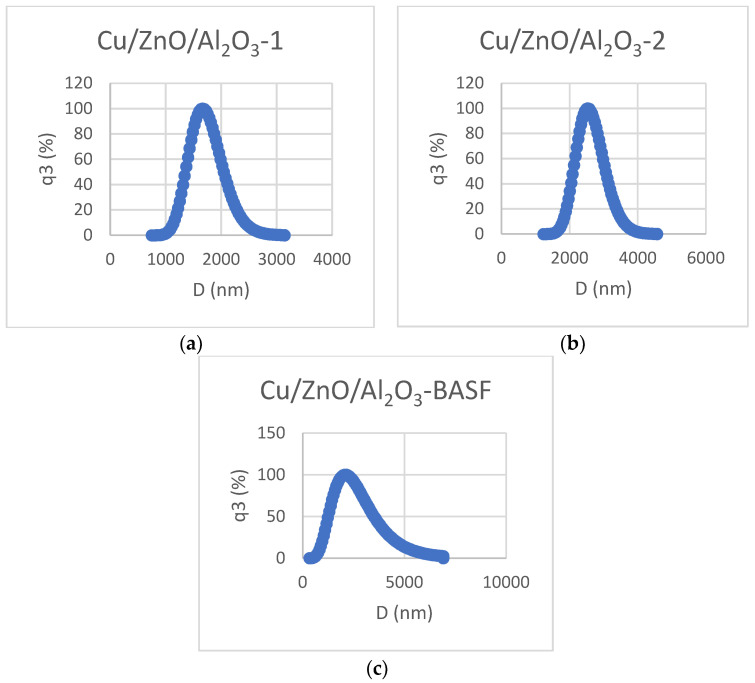
Particle size distribution for Cu/ZnO/Al_2_O_3_-1 (**a**), Cu/ZnO/Al_2_O_3_-2 (**b**), and Cu/ZnO/Al_2_O_3_-BASF (**c**).

The analysis of particle size and its distribution is of paramount importance for determining the properties of particulate materials. The characterization of the catalyst powder nanoparticles was performed by measuring the particle size by Dynamic Light Scattering (DLS) using the Nano DS Dual Light Scattering Particle Size Analyzer (Cilas, Orléans, France). Dynamic light scattering uses Brownian motion to measure the size of nanoparticles. The measurements were performed at a scattering angle of 90° and a temperature of 25 °C with the Cumulant algorithm. The wavelength of the light source was equal to 638 nm. A stable and clean solution was prepared (without sedimentation, agglomeration, aggregation, or flocculation) using 0.002 g of Cu/ZnO/Al_2_O_3_) and 3.998 g of distilled water. The sample vial was placed in an ultrasonic bath for 2 h in order to improve the dispersion of particles. The distribution is monomodal (only one peak, no agglomeration) (Figure 2). The obtained results showed that the particle size of the Cu/ZnO/Al_2_O_3_-1 catalyst ranged between 1373.6 nm and 2139.8 nm, and for the Cu/ZnO/Al_2_O_3_-2 catalyst, the particle size ranged between 2114.3 nm and 3282.3 nm. These results are comparable with the particle size of the Cu/ZnO/Al_2_O_3_-BASF catalyst, which is between 1382.4 nm and 4285.3 nm. In DLS measurements, particle size depends on the translational diffusion coefficient determined thanks to the auto-correlation function. Below are the diagrams of the two samples regarding the particle size distribution.

### 3.3. Determination of Microstructural and Elemental Investigations by Field Emission Scanning Electron Microscope Variable Pressure (FESEM VP) Method

**Figure 3:**
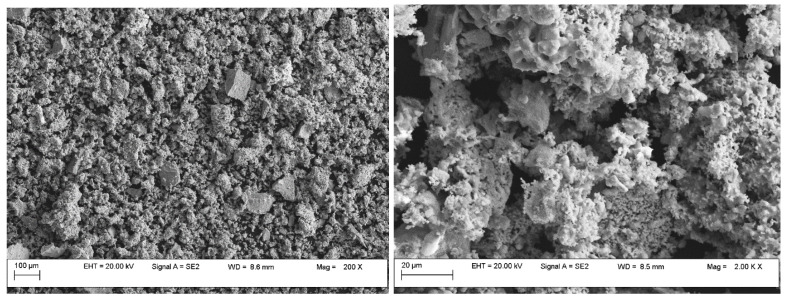
SEM images of Cu/ZnO/Al_2_O_3_-1 catalyst powder.

**Figure 4:**
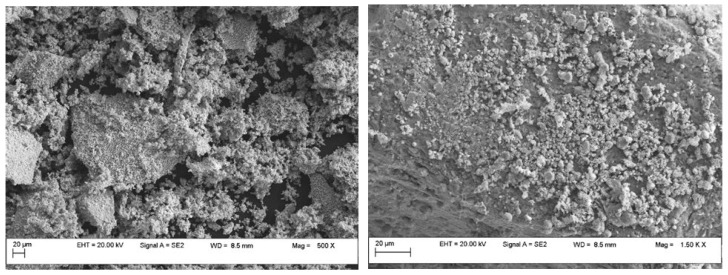
SEM images of Cu/ZnO/Al_2_O_3_-2 catalyst powder.

**Figure 5:**
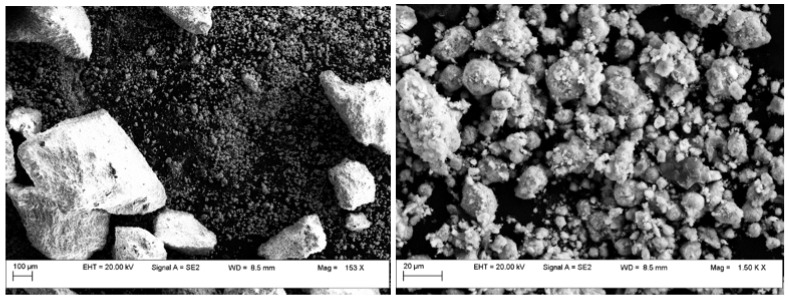
SEM images of Cu/ZnO/Al_2_O_3_—BASF.

**Figure 6:**
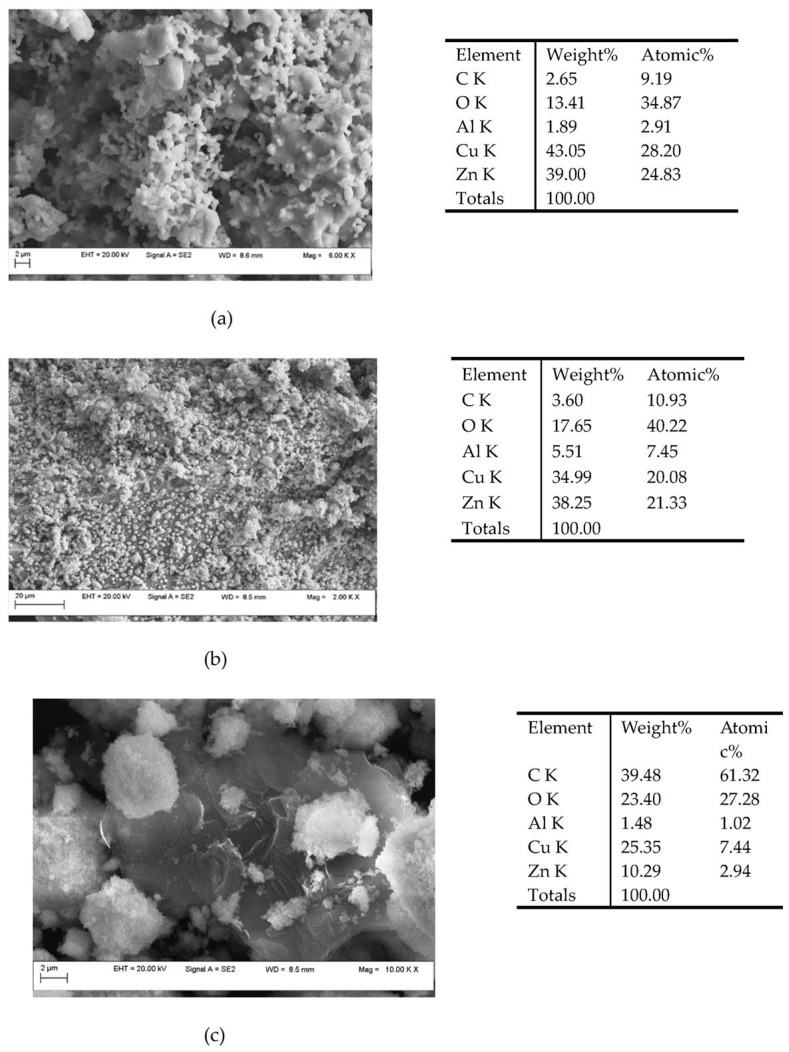
SEM images and EDX analysis of Cu/ZnO/Al_2_O_3_-1 (**a**), Cu/ZnO/Al_2_O_3_-2 (**b**), and Cu/ZnO/Al_2_O_3_-BASF (**c**) powder surfaces.

The microstructural and elemental investigations were carried out using the field emission scanning electron microscope with an auto electronic emission at variable pressure (Field Emission Scanning Electron Microscope Variable Pressure—FESEM VP; brand CARL ZEISS). The microscope is equipped with an EDS (energy dispersive X-ray spectroscopy) system that allows the quantitative and qualitative analysis of all chemical elements up to B (Z = 5). Figure 3 and Figure 4 show the SEM images of Cu/ZnO/Al_2_O_3_-1 and Cu/ZnO/Al_2_O_3_-2 catalyst powder after sintering and Figure 5 for Cu/ZnO/Al_2_O_3_-BASF, which indicate their morphology. The microstructure consists of both micro- and nanometer-size particles and agglomerates. For the microstructural and EDX investigation, the powder was placed on a double-sticky carbon tape, and the surplus was blown with compressed air (Figure 6). The image below represents an SEM micrograph together with the EDX analysis. The particle diameters were in the micron and submicron range.

### 3.4. Determination of Specific Surface Area and Pore Size Distribution by Brunauer–Emmett–Teller (BET) Method

**Figure 7:**
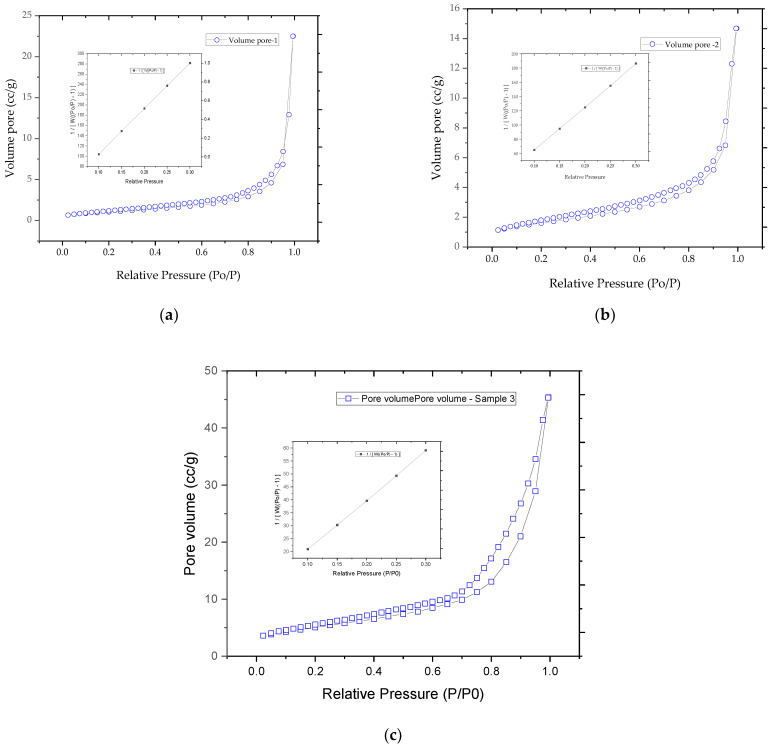
BET adsorption and desorption isotherms, with reference to Cu/ZnO/Al_2_O_3_-1 powder (**a**), Cu/ZnO/Al_2_O_3_-2 (**b**), and Cu/ZnO/Al_2_O_3_-BASF (**c**).

The specific surface area was measured using an Autosorb IQ analyzer (Quantachrome, Boynton Beach, FL, USA) using the BET technique. Nitrogen adsorption–desorption isotherms were obtained at a temperature of 77 K. Samples were degassed prior to analysis at 115 °C for 4–6 h. The specific surface area was calculated using the Brunauer–Emmett–Teller (BET) equation, using the criterion of linearity in the range 0.1–0.3 P/P0. The textural properties of the prepared catalysts were evaluated using the BET method. This is a relevant analysis, especially for the porosity of the prepared catalysts, considering that there must be chemically bound metal centers in a porous support. The pore size distribution was investigated, taking into account the equations corresponding to the theory of the Barrett–Joyner–Halenda (BJH) method using the desorption isotherm. The surface area and microporosity of the Cu/ZnO/Al_2_O_3_ samples were performed by BET and BJH analysis, respectively, and the analysis is shown in Figure 7. The BET analysis is based on the non-selective and reversible physical adsorption between nitrogen gas molecules and the exposed surface of the material at the temperature of liquid nitrogen. The adsorption and desorption isotherms showed a hysteresis loop, suggesting a dominance of the predominantly mesoporous structure; their overlap suggests a relatively weak behavior of the porosity and is included in the field of type IV isotherms. This type refers to the phenomenon of gas condensation in the small capillary pores of the solid material at pressures below their saturation points (capillary condensation), indicating the dominance of finite-length pores; hysteresis arises because of the difference in interference geometry between adsorption and desorption. BJ’s textural analysis is shown in Table 5. Surface analysis was performed using BET analysis (Figure 7). Nitrogen adsorption is caused by the existence of intrinsic surface energies. Cu/ZnO/Al_2_O_3_-1 and Cu/ZnO/Al_2_O_3_-2 powders showed a BET surface of 4.427 m^2^/g and 4.396 m^2^/g, respectively. Nitrogen adsorption/desorption isotherms were measured at 77 K. The obtained curves indicated a hysteresis loop in the adsorption–desorption isotherms, suggesting the presence of a mainly mesoporous structure.

The obtained results showed that the specific BET surfaces of the prepared catalysts had approximately equal values. Therefore, the decisive structural factor in the choice of the catalyst is the pore volume. It is true that the same type of catalysts was used in the methanol production reactions, but catalysts with a copper concentration higher than 40% Cu by weight were used [40].

## 4. Discussion

The main advantage is the method described in this paper. Thus, by a method under mild conditions, starting from different precursors (inorganic and organic), catalysts can be prepared with lower metal concentrations than the commercial ones intended for the same purpose (obtaining methanol) but with similar morphology and pore distribution in the narrowest. field. Another main advantage is derived from the particle distribution. By characterizing the prepared catalysts, the authors of the present study wanted to understand which of the precursors used showed better results. The results of the prepared samples were compared with the results of a commercial catalyst. The results obtained by atomic absorption spectrometry for the prepared catalysts showed that they had similar concentrations of copper (29.2 % Cu for Cu/ZnO/Al_2_O_3_-1, respectively 27.3 % Cu for Cu/ZnO/Al_2_O_3_-2). Thus, the commercial catalyst has a value between 1382.4 nm and 4285.3 nm, and the prepared catalysts have between 1373.6 nm and 2139.8 nm for the catalyst Cu/ZnO/Al_2_O_3_-1, and between 2114.3 nm and 3282.3 nm for catalyst Cu/ZnO/Al_2_O_3_-2. The pore distribution evaluated by the BET and BJH methods is also noteworthy. The SEM images of the powder of the prepared catalysts, after sintering, indicated micro- and nano meter sized particles, but also agglomerates. The surface of the powder of the prepared catalysts, using the BET technique, showed that the adsorption and desorption isotherms presented a hysteresis loop, suggesting the dominance of the predominantly mesoporous structure. Their overlap suggests a relatively weak behaviour of the porosity and is included in the field of type IV isotherms. Following these investigations, it appears that the best results were obtained for the catalyst with an inorganic precursor.

In the literature, for the preparation of catalysts based on copper and zinc, they contain copper nitrate mixed with zinc nitrate, impregnated on alumina and its characterization is presented in the Table 6.

## 5. Conclusions

This paper presents the synthesis of alternative copper-based catalysts for the production of methanol under mild reaction conditions, by the impregnation method. Therefore, in the following study we will evaluate the catalytic activity of catalysts with a concentration of 30% Cu and, concretely, the present study presents the stages of their preparation and characterization. Future studies will consist of using the obtained catalysts in the production of methanol. The methanol obtained will be used in direct methanol fuel cells. The direct methanol fuel cell (DMFC) is an electrochemical energy source based on a simple principle, but presents a complexity regarding the implementation of energy generation systems in commercial applications. Fuel Cell is the market leader in fuel cell systems for off-grid and mobile applications such as mission critical communications, information technology, optronics, sensors and auxiliary power.

## Figures and Tables

**Table 1:** The method of preparation of the catalysts.

Catalyst	Precursor	Preparation of Catalyst
Cu/ZnO/Al_2_O_3_-1	Copper nitrate	- 40 g copper nitrate; - 5 g Al_2_O_3_; - 20 g ZnO; - 200 mL distilled water
Cu/ZnO/Al_2_O_3_-2	Copper acetate	- 40 g copper acetate; - 5 g Al_2_O_3_; - 20 g ZnO; - 200 mL distilled water

**Table 2:** Mineralization program.

Step	Power (W)	Power (%)	Ramp (min)	Pression (PSI)	Temperature (°C)
1	400	1000	10	350	160
2	400	100	10	350	160
3	Cooling				

**Table 3:** Instrumental parameters atomic absorption spectrophotometer in the flame.

Parameters	Element	
	Cu	Zn
Wavelength (nm))	324.8	213.8
Lamp current intensity (mA)	5.0	6.0
Working range (mg·l^−1^)	0.2–8.0	0.05–1.00

**Table 4:** The results of the samples prepared by AAS analysis.

Sample	%Cu	%Zn
Cu/ZnO/Al_2_O_3_-1	29.2	31.5
Cu/ZnO/Al_2_O_3_-2	27.3	32.3
Cu/ZnO/Al_2_O_3_-BASF	41.2	14.4

**Table 5:** Specific surface analysis (BET method) ^1^ and porosity (BJH method) ^2^ for prepared and commercial catalysts.

Sample	Specific Surface Area ^1^ (m^2^ g^−1^)	Pore Volume ^2^ (cm^3^ g^−1^)	Average Pore Radius ^2^ (A)
Cu/ZnO/Al_2_O_3_-1	4.427	0.035	15.650
Cu/ZnO/Al_2_O_3_-2	4.396	0.021	19.650
Cu/ZnO/Al_2_O_3_-BASF	77.927	0.222	18.516

^1^ Measured by BET method; ^2^ Measured by BJH method.

**Table 6:** Catalyst characterization reported in the literature.

Sample	Method of Catalyst Preparation	Cu:Zn:Al (%w/w) Mass Ratio	Specific Surface Area ^1^ (m^2^ g^−1^)	Pore Volume ^2^ (cm^3^ g^−1^)	Average Pore Radius^2^ (A)	References
Cu/ZnO/Al_2_O_3_-1	Impregnation	29.2:31.5:00	4.427	0.035	15.650	Present Work
Cu/ZnO/Al_2_O_3_-2		27.3:32.3:00	4.396	0.021	19.650	
Cu/ZnO/Al_2_O_3_-BASF	-	41.2:14.4:00	77.927	0.222	18.516	
Cu/ZnO/Al_2_O_3_	Impregnation	50:50:00	58.24	0.436	299.1	[41]
Cu/ZnO/Al_2_O_3_	Impregnation	75:25:00	36.11	0.241	266.6	[41]
Cu/ZnO/Al_2_O_3_	Impregnation	60:30:10	77.02	0.578	300.3	[41]
Cu/ZnO/Al_2_O_3_	Impregnation	3:3:0	67.6	-	-	[42]
Cu/ZnO/Al_2_O_3_	Co-precipitation	60:30:10	42.9	0.23	-	[43]

^1^ Measured by BET method; ^2^ Measured by BJH method.

## Data Availability

Data are contained within the article.

## References

[1] Borisut P., Nuchitprasittichai A. (2019). Methanol production via CO_2_ hydrogenation: Sensitivity analysis and simulation—Based optimization. Front. Energy Res..

[2] Grabow L.C., Mavrikakis M. (2011). Mechanism of methanol synthesis on Cu through CO_2_ and CO hydrogenation. Acs Catal..

[3] Deka T.J., Osman A.I., Baruah D.C., Rooney D.W. (2022). Methanol fuel production, utilization, and techno-economy: A review. Environ. Chem. Lett..

[4] Marlin D.S., Sarron E., Sigurbjörnsson Ó. (2018). Process advantages of direct CO_2_ to methanol synthesis. Front. Chem..

[5] Olah G.A., Prakash G.K.S., Goeppert A. (2011). Anthropogenic Chemical Carbon Cycle for a Sustainable Future. J. Am. Chem. Soc..

[6] Zhong J., Yang X., Liang Z., Huang B., Zhang Y. (2020). State of the art and perspectives in heterogeneous catalysis of CO_2_ hy-drogenation to methanol. Chem. Soc. Rev..

[7] Zhao B., Li C., Hu T., Zhang X. (2023). Nanoporous {Pb3}-Organic Framework for Catalytic Cycloaddition of CO_2_ with Epoxides and Knoevenagel Condensation. ACS Appl. Nano Mater..

[8] Li C., Lv H., Yang K., Zhang X. (2023). Robust fluorine-functionalized {Ln5}-organic frameworks for excellent catalytic performance on cycloaddition of CO_2_ with epoxides and knoevenagel condensation. ACS Appl. Mater. Interfaces.

[9] Shen W.J., Jun K.W., Choi H.S., Lee K.W. (2000). Thermodynamic investigation of methanol and dimethyl ether synthesis from CO_2_ hydrogenation. Korean J. Chem. Eng..

[10] Ipatieff V.N., Monroe G.S. (2002). Synthesis of methanol from carbon dioxide and hydrogen over Copper-Alumina catalysts. mechanism of reaction. J. Am. Chem. Soc..

[11] Boretti A. (2013). Renewable hydrogen to recycle CO_2_ to methanol. Int. J. Hydrogen Energy.

[12] Jiang X., Nie X., Guo X., Song C., Chen J.G. (2020). Recent advances in carbon dioxide hydrogenation to methanol via hetero-geneous catalysis. Chem. Rev..

[13] Riaz A., Zahedi G., Klemeš J.J. (2013). A review of cleaner production methods for the manufacture of methanol. J. Clean. Prod..

[14] Quadrelli E.A., Centi G., Duplan J.L., Perathoner S. (2011). Carbon dioxide recycling: Emerging large-scale technologies with in-dustrial potential. ChemSusChem.

[15] Dang S., Yang H., Gao P., Wang H., Li X., Wei W., Sun Y. (2019). A review of research progress on heterogeneous catalysts for methanol synthesis from carbon dioxide hydrogenation. Catal. Today.

[16] Guil-López R., Mota N., Llorente J., Millán E., Pawelec B., Fierro J.L.G., Navarro R.M. (2019). Methanol synthesis from CO_2_: A review of the latest developments in heterogeneous catalysis. Materials.

[17] Berahim N.H., Zabidi N.A.M. (2022). Catalytic conversion of CO_2_ into methanol. Carbon Dioxide Capture and Conversion.

[18] Rama M., Rowley C. (2017). Changing the Indian Economy.

[19] Védrine J.C. (2017). Heterogeneous catalysis on metal oxides. Catalysts.

[20] Thommes M., Kaneko K., Neimark A.V., Olivier J.P., Rodriguez-Reinoso F., Rouquerol J., Sing K.S. (2015). Physisorption of gases, with special reference to the evaluation of surface area and pore size distribution (IUPAC Technical Report). Pure Appl. Chem..

[21] Liu G., Hagelin-Weaver H., Welt B. (2023). A concise review of catalytic synthesis of methanol from synthesis gas. Waste.

[22] Huang L., Kramer G.J., Wieldraaijer W., Brands D.S., Poels E.K., Castricum H.L., Bakker H. (1997). Methanol synthesis over Cu/ZnO catalysts prepared by ball milling. Catal. Lett..

[23] Burch R., Chappell R.J. (1988). Support and additive effects in the synthesis of methanol over copper catalysts. Appl. Catal..

[24] Bowker M. (2019). Methanol synthesis from CO_2_ hydrogenation. ChemCatChem.

[25] Behrens M., Studt F., Kasatkin I., Kühl S.M., Hävecker F., Abild-Pedersen S., Zander F., Girgsdies P., Kurr B.-L., Kniep M. (2012). The Active Site of Methanol Synthesis over Cu/ZnO/Al_2_O_3_ Industrial Catalysts. Science.

[26] Mota N., Guil-Lopez R., Pawelec B.G., Fierro J.L.G., Navarro R.M. (2018). Highly active Cu/ZnO–Al catalyst for methanol synthesis: Effect of aging on its structure and activity. RSC Adv..

[27] Ghosh S., Uday V., Giri A., Srinivas S. (2019). Biogas to methanol: A comparison of conversion processes involving direct carbon dioxide hydrogenation and via reverse water gas shift reaction. J. Clean. Prod..

[28] Kondrat S.A., Smith P.J., Wells P.P., Chater P.A., Carter J.H., Morgan D.J., Hutchings G.J. (2016). Stable amorphous georgeite as a precursor to a high-activity catalyst. Nature.

[29] Kühl S., Tarasov A., Zander S., Kasatkin I., Behrens M. (2014). Cu-Based Catalyst Resulting from a Cu, Zn, Al Hydrotalcite-Like Compound: A Microstructural, Thermoanalytical, and In Situ XAS Study. Chem. A Eur. J..

[30] Guil-López R., Mota N., Llorente J., Millán E., Pawelec B., García R., Fierro J.L.G. (2019). Data on TGA of precursors and SEM of reduced Cu/ZnO catalysts co-modified with aluminium and gallium for methanol synthesis. Data Brief.

[31] Fujita S.I., Moribe S., Kanamori Y., Kakudate M., Takezawa N. (2001). Preparation of a coprecipitated Cu/ZnO catalyst for the methanol synthesis from CO_2_—Effects of the calcination and reduction conditions on the catalytic performance. Appl. Catal. A Gen..

[32] Behrens M., Brennecke D., Girgsdies F., Kißner S., Trunschke A., Nasrudin N., Schlögl R. (2011). Understanding the complexity of a catalyst synthesis: Co-precipitation of mixed Cu, Zn, Al hydroxycarbonate precursors for Cu/ZnO/Al_2_O_3_ catalysts investi-gated by titration experiments. Appl. Catal. A Gen..

[33] Jeong Y., Kim I., Kang J.Y., Jeong H., Park J.K., Park J.H., Jung J.C. (2015). Alcohol-assisted low temperature methanol synthesis from syngas over Cu/ZnO catalysts: Effect of pH value in the co-precipitation step. J. Mol. Catal. A Chem..

[34] Smith P.J., Kondrat S.A., Chater P.A., Yeo B.R., Shaw G.M., Lu L., Hutchings G.J. (2017). A new class of Cu/ZnO catalysts derived from zincian georgeite precursors prepared by co-precipitation. Chem. Sci..

[35] Huang L., Chu W., Long Y., Ci Z., Luo S. (2006). Influence of Zirconia Promoter on Catalytic Properties of Cu–Cr–Si Catalysts for Methanol Synthesis at High CO Conversion in Slurry Phase. Catal. Lett..

[36] Chu W., Zhang T., He C., Wu Y. (2002). Low-temperature methanol synthesis (LTMS) in liquid phase on novel copper-based cat-alysts. Catal. Lett..

[37] Mashayekh-Salehi A., Moussavi G., Yaghmaeian K. (2017). Preparation, characterization and catalytic activity of a novel mesopo-rous nanocrystalline MgO nanoparticle for ozonation of acetaminophen as an emerging water contaminant. Chem. Eng. J..

[38] Shi L., Tao K., Yang R., Meng F., Xing C., Tsubaki N. (2011). Study on the preparation of Cu/ZnO catalyst by sol–gel au-to-combustion method and its application for low-temperature methanol synthesis. Appl. Catal. A Gen..

[39] Pori M., Arčon I., Dasireddy V.D., Likozar B., Orel Z.C., Marinšek M. (2021). Photo-Chemically-Deposited and Industrial Cu/ZnO/Al_2_O_3_ Catalyst Material Surface Structures During CO_2_ Hydrogenation to Methanol: EXAFS, XANES and XPS Analyses of Phases after Oxidation, Reduction, and Reaction. Catal. Lett..

[40] Ahouari H., Soualah A., Le Valant A., Pinard L., Magnoux P., Pouilloux Y. (2013). Methanol synthesis from CO_2_ hydrogenation over copper based catalysts. React. Kinet. Mech. Catal..

[41] Prasetyaningsih Y., Hendriyana H., Susanto H. (2016). Influence of impregnation and coprecipitation method in preparation of Cu/ZnO catalyst for methanol synthesis. J. Eng. Technol. Sci..

[42] Wang L., Etim U.J., Zhang C., Amirav L., Zhong Z. (2022). CO_2_ Activation and Hydrogenation on Cu-ZnO/Al_2_O_3_ Nanorod Catalysts: An In Situ FTIR Study. Nanomaterials.

[43] Kipnis M., Volnina E., Belostotsky I., Galkin R., Zhilyaeva N., Levin I., Ezhov A. (2022). Effective Cu/ZnO/Al_2_O_3_ Catalyst for Methanol Production: Synthesis, Activation, Catalytic Performance, and Regeneration. Catal. Res..

